# Preoperative platelet to lymphocyte and neutrophil to lymphocyte ratios are independent prognostic factors for patients undergoing lung cancer radical surgery: A single institutional cohort study

**DOI:** 10.18632/oncotarget.13312

**Published:** 2016-11-11

**Authors:** Haidan Lan, Leng Zhou, Dongmei Chi, Qinghua Zhou, XiaoJun Tang, Daxing Zhu, Jianmin Yue, Bin Liu

**Affiliations:** ^1^ Department of Anesthesiology, West China Hospital of Sichuan University, Chengdu, Sichuan, China; ^2^ The lung cancer center, West China Hospital of Sichuan University, Chengdu, Sichuan, China

**Keywords:** non small cell lung cancer, platelet to lymphocyte ratio, neutrophil to lymphocyte ratio, postoperative pulmonary complications, overall survival

## Abstract

**Background:**

The aim of this study was to assess the prognostic value for NSCLC patients who were scheduled to receive lung cancer radical resection.

**Methods:**

In this cohort study (Dec.2014-Feb.2016), patients with non-small cell lung cancer (NSCLC) who underwent radical lung cancer thoracotomy were enrolled and accessed at postoperative complications, one-year overall survival (OS) and relapse-free survival (RFS). The preoperative PLR and NLR of all patients were calculated based on preoperative complete blood counts. Univariate and multivariate Cox regression analyses were performed to determine the associations of PLR and NLR with OS and RFS.

**Results:**

A total of 174 NSCLC patients were studied. The results indicated that both high PLR (>148.6) and NLR (>2.9) were related to a high rate of postoperative pulmonary complications significantly (49.3%vs.29.1%, *P* = 0.007; 50.7% *vs*. 28.6%, *P* = 0.003). Moreover, NSCLC patients with a high PLR level (> 148.6) was significantly associated with a lower one-year OS (90.3% *vs*. 77.5%, *P* = 0.034).

**Conclusions:**

Preoperative PLR and NLR were good prognostic factors for postoperative pulmonary complications and OS in NSCLC patients undergoing radical lung cancer surgery. Thus, blood PLR and NLR would be helpful as a prognostic tool before radical lung cancer surgery.

## INTRODUCTION

Lung cancer is the most common malignancy, and it is also the leading cause of cancer death worldwide [1]. The primary treatment measure for lung cancer is radical lung cancer surgery [2, 3]. Although great advance in lung cancer surgery has developed, the prognosis of lung cancer is still unsatisfactory. Studies reported that the median survival time for lung cancer in China is only 22.7 months [2]. Until now, ideal method to evaluate the prognosis of lung cancer remains unavailable. Thus, it is urgent and essential to identify a reliable prognostic marker for patients who were scheduled to receive lung cancer surgery. It would help clinicians to make good risk stratification and choose individualized therapy strategies for lung cancer patients.

In lung cancer radical thoracotomy, the preoperative immune status has a close relationship with clinical outcomes according to recent studies [4, 5]. Besides, accumulating evidence has shown that the development and progression of tumor were associated with systemic inflammation because a cross-talk between inflammation and cancer might occur during perioperative period [6, 7]. As the conceptual tumor develops over last decade, evading immune destruction has become a cancer progress hallmark [6]. Systemic inflammatory response plays an important role in cancer development or progression but different inflammation-based indices account for different type of cancer [8, 9]. Theoretically, inflammatory status should convey valuable information of lung cancer development or progression but unfortunately most specific inflammatory factors are not accessible before surgery and some are only available as research tools.

Preoperative platelet to lymphocyte ratio (PLR) and neutrophil to lymphocyte ratio (NLR) are two simply and widely available markers to reflect systemic inflammation status [10]. These two markers have been shown to be prognostic for several cancer types such as gastric, colorectal, liver and pancreatic cancers [9, 11–13]. Increased circulating platelets may contribute to tumor cell emboli and finally accelerate the course of tumor progression. Surgery promotes the formation of platelet clots around tumor cell emboli, thereby impairing NK cell-mediated tumor cell clearance, whereas perioperative anticoagulation attenuates this effect [14]. Preoperative high NLR relate to increased neutrophil-dependent inflammation, and reduced lymphocyte-mediated tumor response. Neutrophils may be recruited to lung carcinomas to enhance the invasive and metastatic potential of lung cancer cells [15]. An excess of neutrophil elastase is significantly associated with lung cancer risk [16]. Moreover, neutrophils can favor tumor development and inhibit the activity of lymphocytes and other immune cells. All the factors mentioned above is closely related to prognosis of lung cancer radical surgery. However, few prospective studies have investigated the association between preoperative PLR or NLR level and NSCLC clinical outcomes including postoperative pulmonary complications and one-year overall survival. Therefore, we conducted a prospective observational study to investigate the relationship between PLR or NLR and early clinical outcomes or one-year survival in NSCLC patients after lung cancer radical surgery.

## RESULTS

### Patient characteristics

The demographic and clinical characteristics of study population are summarized in Table 1. Between December 2014 and February 2016, a total of 435 patients were screened of whom 186 were enrolled finally and 174 (95.38%) were confirmed with NSCLC based on postoperative pathology. The mean age of the 174 patients was 59.02 years (SD 11.33) with 122 (70.1%) male patients. The most common initial clinical symptom was cough and expectoration (43.1%). Almost a quarter of patients were found lung cancer through health examination without any clinical symptom. Only 20 percent people had adjuvant therapy before surgery.

**Table 1:** Demographic and clinical characteristics of study population

Characteristics	Value
Subjects	174
Age, years	59.02±11.33
Gender	
Male	122(70.1%)
Female	52(29.9%)
Body-mass index, kg/m^2^	23.1±2.90
Smoking history	
Never	64(36.8%)
Smoker	110(63.2%)
ASA classification	
I	11(6.3%)
II	125(71.8%)
III	38(21.8%)
Lung function	
FVC (L)	3.07±0.80
FEV1 (L)	2.30±0.64
FEV1/FVC (%)	74.10±10.80
Comorbidities	
Hypertension	28(16.1%)
Diabetes mellitus	14(8.1%)
Heart disease	25(14.4%)
COPD	35(20.1%)
Clinical symptoms	
No	40(23.0%)
Cough or expectoration,	75(43.1%)
Haemoptysis	28(16.1%)
Chest pain	19(10.9%)
Others	12(6.9%)
Adjuvant therapy	35(20.1%)
AFP (ng/mL)	6.50±30.98
CEA (ng/mL)	20.03±66.87
CA199 (U/mL)	20.78±32.33
CA125 (U/mL)	117.92±824.68
PLR	157.11±87.95
NLR	2.82±1.58

Data are presented as n(%) or mean ± standard differences (SD).

COPD: chronic obstructive pulmonary disease; ASA: American society of anesthesiologists; FEV1: forced expiratory volume in one second; AFP: alpha fetal protein; CEA: carcino embryonie antigen; PLR: platelet to lymphocyte ratio; NLR: neutrophil to lymphocyte ratio.

### Association between the PLR or NLR and clinicopathological characteristics

The association between preoperative PLR or NLR and clinicopathological characteristics is shown in Table 2. The patients’ preoperative PLR values distributed evenly among clinicopathological parameters, except for pathological type. Patients with squamous cell cancer have a higher PLR than adenocarcinoma (*P* = 0.006, Table 2). The preoperative NLR was significantly higher in patients with the following characteristics: male (*P* = 0.001), haemoptysis (*P* = 0.014), smoker (*P* = 0.010), squamous carcinoma (*P* = 0.012) (Table 2).

**Table 2:** The relationship between PLR, NLR and clinicopathological characteristics

	*N*=174	PLR	*P* value	NLR	*P* value
Age, year					
≤ 60	88(50.6%)	155.55±70.62	0.557	2.77±1.46	0.873
> 60	86(49.4%)	148.87±79.04		2.73±1.37	
Gender					
Male	122(70.1%)	160.92±91.50	0.387	3.09±1.64	0.001*
Female	52(29.9%)	148.26±79.22		2.24±1.27	
Clinical symptoms					
No	40(23.0%)	127.44±47.63	0.117^#^	2.21±0.88	0.014*^#^
Cough or expectoration	75(43.1%)	154.56±73.66		2.74±1.35	
Haemoptysis	28(16.1)	168.30±85.53		3.40±1.36	
Chest pain	19(10.9)	173.73±85.58		2.95±1.84	
Others	12(6.9%)	149.01±98.37		2.76±1.98	
Smoking history					
Never	64(36.8%)	157.99±79.69	0.441	2.39±1.22	0.010*
Smoker	110(63.2%)	148.90±71.90		2.96±1.48	
COPD history					
YES	35(20.1%)	156.09±93.48	0.939	3.10±1.65	0.267
NO	139(79.9%)	157.37±86.85		2.76±1.56	
Type of resection					
Sublobectomy	11(6.3%)	140.52±114.86	0.872^#^	2.68±2.35	0.942^#^
Lobectomy	128(73.6%)	150.94±67.87		2.72±1.26	
Biolectomy	28(16.1%)	153.98±79.80		2.90±1.74	
Peneumonectomy	7(4.0%)	179.04±103.00		2.81±1.01	
Sleeve lobectomy					
YES	41(23.6%)	146.88±54.55	0.600	2.90±1.44	0.454
NO	133(76.4%)	153.90±80.07		2.71±1.41	
Tumor location					
Left	70(40.2%)	157.98±78.53	0.408	2.89±1.30	0.278
Right	104(59.8%)	148.39±72.24		2.66±1.47	
Pathological type					
AC	64(36.8%)	137.51±64.82	0.006*^#^	2.48±1.31	0.012*^#^
SCC	96(55.2%)	165.21±74.96		3.15±1.48	
Others	14(8.0%)	193.98±110.14		2.75±1.42	
Pathological stage					
I	29(16.7%)	135.55±42.24	0.280^#^	2.44±1.057	0.380^#^
II	34(19.5%)	172.87±92.20		3.06±1.38	
III	88(50.6%)	152.15±86.98		2.77±1.64	
IV	23(13.2%)	169.58±98.34		3.05±1.93	

*P < 0.05 is significant. Data are presented as n(%) or mean±standard differences.

Data are compared by student t test or # by one-way ANOVA (Analysis of Variance)

AC: adenocarcinoma; SCC: squamous carcinoma.

### Receiver operating characteristic curve of PLR and NLR for OS prediction

**Figure 1:**
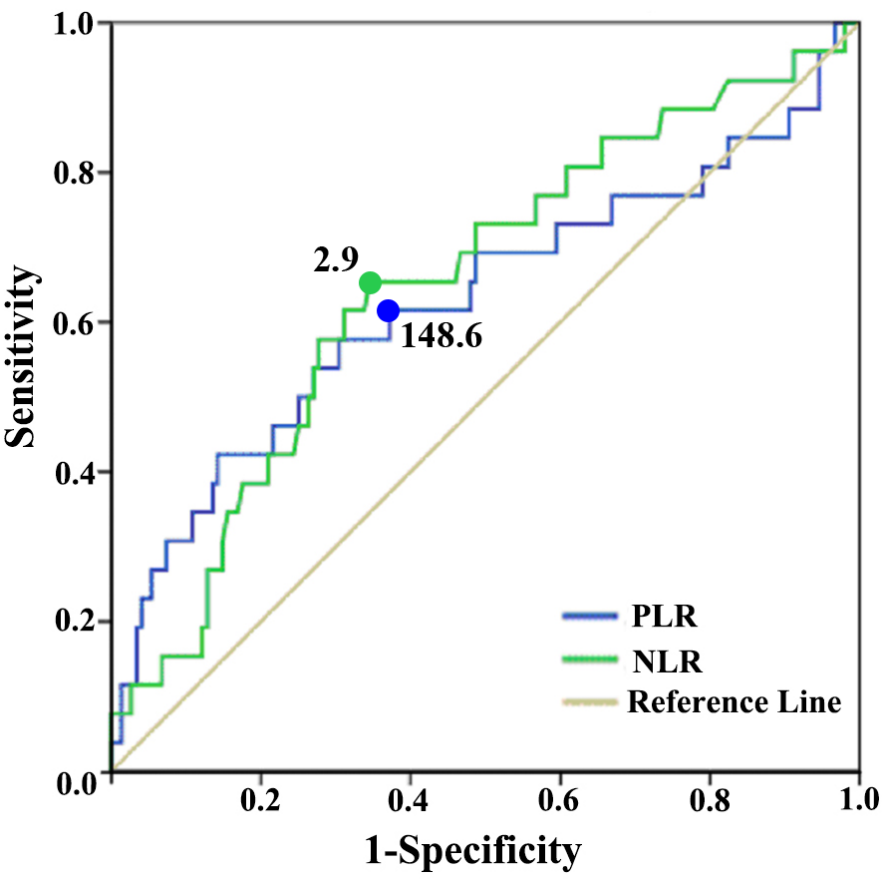
ROC curves for survival predication ROC curves were potted to verify the accuracy of PLR and NLR for survival. The area under the curve (AUC) was 0.628 (*P* = 0.037, 0.493-0.763) for PLR, and 0.648(*P* = 0.016, 0.531-0.766) for NLR.

The optimal cutoff values for PLR and NLR to predict OS were identified by the receiver operating characteristic (ROC) curve (Figure 1). They were 148.6 for PLR and 2.9 for NLR. Besides, the area under the curve (AUC) for PLR and NLR were 0.628 (*P* = 0.037, 95%CI = 0.493-0.763) and 0.648 (*P* = 0.016, 95%CI = 0.531-0.766 ), separately (Figure 1). Based on the cut-off value, a total of 71 patients (40.8%) were detected with high preoperative PLR ( > 148.6, Table 2) and in the same way 69 patients (39.7%) had high preoperative NLR ( > 2.9, Table 2).

### Association between the PLR or NLR and postoperative clinical outcomes

The postoperative clinical outcomes were compared between patients according to their PLR or NLR status through χ2 test or Fisher's exact test and the results were showed in Table 3. High PLR(>148.6) was significantly correlated with high rate of pneumonia (*P* = 0.006), pulmonary complications (*P* = 0.007), length of postoperative hospital stay (*P* = 0.002) and poor wound healing (*P* = 0.013). The High level of PLR did not correlate with respiratory failure (*P* = 0.938), ARDS (*P* = 0.577) or atelectasis (*P* = 0.085). When it comes to NLR, high NLR(>2.9) significantly associate with high rate of pneumonia (*P* = 0.049); pulmonary complications (*P* = 0.003), length of postoperative hospital stay(*P* = 0.032) and postoperative atelectasis (*P* < 0.001, Table 3). High NLR had no association with respiratory failure (*P* = 0.973) and extra-pulmonary complications (Table 3).

**Table 3:** The relationship between PLR, NLR and postoperative complications

	PLR			NLR		
	≤148.6	>148.6	*P* value	≤2.9	>2.9	*P* value
Number	103(59.2%)	71(40.8%)	-	105(60.3%)	69(39.7%)	-
Pulmonary complications						
Pneumonia	22(21.4%)	29(40.8%)	0.006*	25(23.8%)	26(37.7%)	0.049*
Bronchopleural fistula	2(1.9%)	2(2.8%)	0.705^#^	3(2.9%)	1(1.4%)	0.929^#^
Respiratory failure	12(11.7%)	8(11.3%)	0.938	12(11.4%)	8(11.6%)	0.973
ARDS/ALI	21(20.4%)	17(23.9%)	0.577	20(19.0%)	18(26.1%)	0.027*
Atelectasis	13(12.6%)	16(22.5%)	0.085	8(7.6%)	21(30.4%)	<0.001*
Any	30(29.1%)	35(49.3%)	0.007*	30(28.6%)	35(50.7%)	0.003*
Extra-Pulmonary complications						
Arrhythmia	14(13.6%)	11(15.5%)	0.725	11(10.05%)	14(20.3%)	0.071
Cardiac failure	4(3.9%)	4(5.6%)	0.717^#^	6(5.7%)	2(2.9%)	0.619^#^
Poor wound healing	7(6.8%)	13(18.3%)	0.013*	9(8.6%)	11(15.9%)	0.136
Prolonged airleak	16(15.5%)	17(23.9%)	0.164	17(16.2%)	16 (23.2%)	0.249
Chest drainage(ml)	820±688	860±597	0.708	792±672	901±623	0.315
Hospital mortality	2(1.9%)	2(2.8%)	0.540^#^	3(2.9%)	1(1.4%)	0.480^#^
Postoperative hospital stay (days)	11.45±3.43	13.47±4.56	0.002*^&^	11.71±3.73	13.13±4.36	0.032*^&^

*P < 0.05 is significant

Data are presented as n (%) or mean ± standard differences.

Data are compared by χ2 test or # by Fisher's exact test; & By student *t* test

ARDS: Acute Respiratory Distress Syndrome; ALI: Acute Lung Injury

### Association of the PLR and NLR with survival

**Figure 2:**
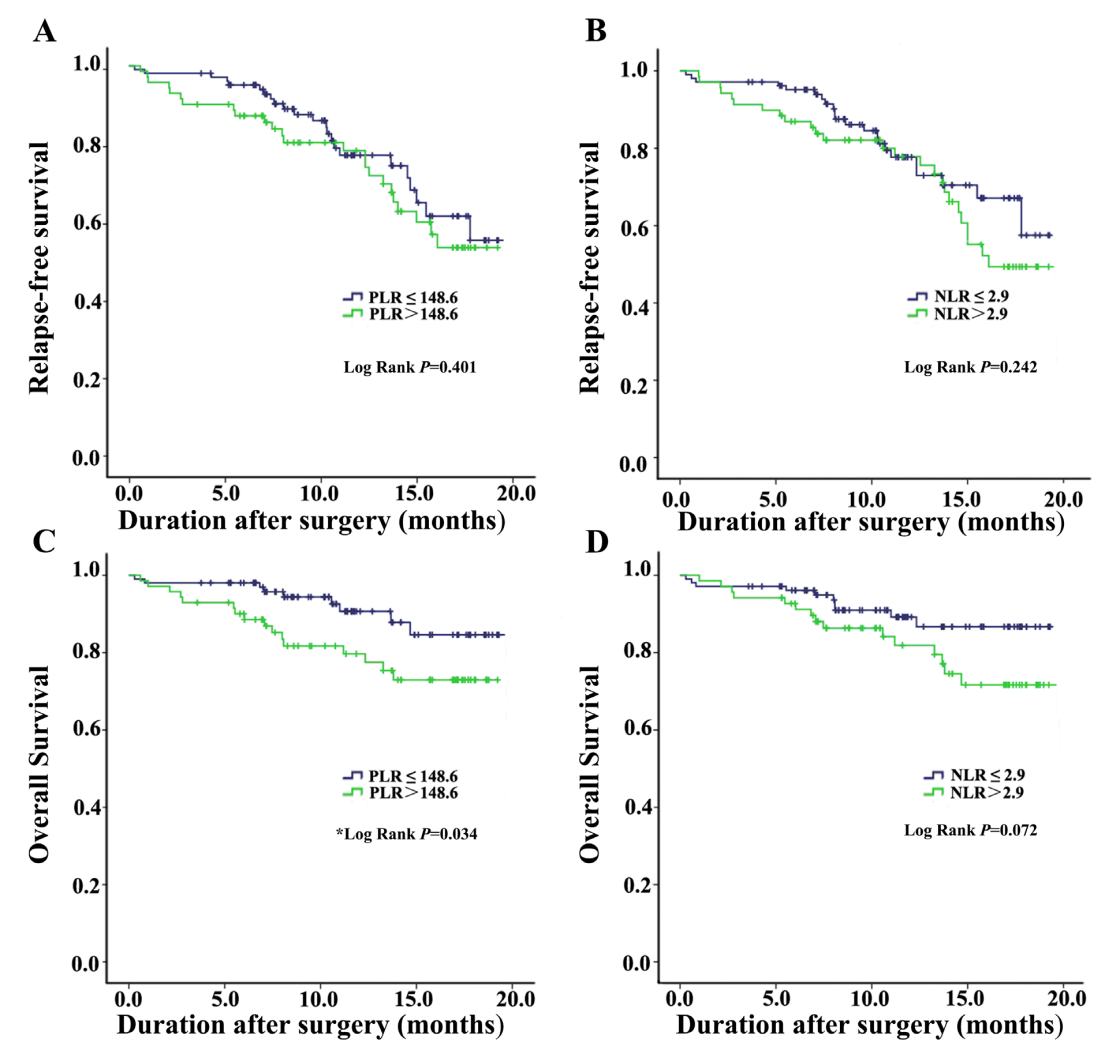
Kaplan-Meier curves estimates for the effect of pretreatment PLR, PLR on RFS and OS of NSCLC patients **A**. Relapse-free survival curves at one year (Kaplan-Meier analysis) for NSCLC patients with PLR≤148.6 or > 148.6. **B**. Relapse-free survival curves at one year (Kaplan-Meier analysis) for NSCLC patients with NLR≤2.9 or > 2.9. **C**. Overall survival curves at one year (Kaplan-Meier analysis) for NSCLC patients with PLR≤148.6 or > 148.6. **D**. Overall survival curves at one year (Kaplan-Meier analysis) for NSCLC patients with NLR≤2.9 or > 2.9. *P < 0.05 is significant

The median follow-up duration was 11 months (IOR, 7-16). Patients with high PLR had a worse one-year OS than those with low PLR (90.3% vs. 77.5%, *P* = 0.034) (Figure 2C). Different from PLR, no significant difference was investigated between groups concerning NLR (90.5% vs. 76.8%, *P* = 0.072). However, a potential trend between high NLR and a worse OS remained to be seen from the ROC curve (Figure 2D). There were no significantly difference observed in relapse-free survival between different level of PLR or NLR (*P* = 0.401, *P* = 0.242, Figure 2A, 2B).

### Cox regression analysis

The results of univariate and multivariate analyses for survival were shown in Table 4 and Table 5. On univariate analysis, high pathological stage (hazard ratio (HR) 3.36; 95% CI 1.15-9.79, *P* = 0.026, Table 4), PLR (HR:1.06 95% CI: 1.02-1.10, *P* = 0.002, Table 4) and NLR (HR:1.37 95% CI 1.08-1.73, *P* < 0.010, Table 4) were significant prognostic factors for poor OS. Meanwhile high stage (III/IV) of NSCLC (HR:2.20; 95% CI 1.12-4.32, *P* < 0.022) and PLR (HR:1.04; 95% CI 1.01-1.07, *P* < 0.017, Table 4) were associated with poor RFS. No significance difference was observed between preoperative NLR and RFS (*P* = 0.162, Table 4). Multivariate analysis identified pathological stage (HR: 4.08; 95%CI 1.31-12.73, *P* = 0.015) as an independent poor prognostic factor for OS and PLR (HR 1.06); 95% CI 1.01-1.11; *P* = 0.023) as an independent poor prognostic factor for RFS (Table 5). There was no significant association between PLR (P = 0.103) or NLR (*P* = 0.819) and NSCLC patients’ one-year OS.

**Table 4:** univariate Cox regression analysis for PLR and NLR of RFS and OS

	Overall survival		Progression-free survival	
	HR(95%CI)	*P* value	HR(95%CI)	*P* value
Age(≤60, >60)	0.96(0.40-2.30)	0.927	1.27(0.69-2.32)	0.444
Gender (Male/ Female)	2.26(0.93-5.50)	0.084	1.41(0.73-2.71)	0.310
Smoke history (yes, no)	1.32(0.58-3.05)	0.523	1.06(0.59-1.93)	0.841
COPD	1.31(0.52-3.29)	0.561	1.41(0.73-2.72)	0.304
Pathological stage				
(I/II)/ (III/IV)	3.36(1.15-9.79)	0.026*	2.20(1.12-4.32)	0.022*
Pathological type				
AC	1		1	
SCC	1.11(0.47-2.65)	0.805	0.91(0.49-1.67)	0.753
Others	2.85(0.40-3.32)	0.071	1.16(0.40-3.33)	0.786
PLR	1.06(1.02-1.10)	0.002*	1.04(1.01-1.07)	0.017*
NLR	1.37(1.08-1.73)	0.010*	1.14(0.95-1.38)	0.162

*P < 0.05 is significant

HR: hazard rate; 95% CI: 95% confidence interval;

**Table 5:** multivariate Cox regression analysis for PLR and NLR of RFS and OS

	Overall survival		Progression-free survival	
	HR(95%CI)	*P* value	HR(95%CI)	*P* value
Age(≤60, >60)	1.63(0.59-4.46)	0.345	1.86(0.917-3.77)	0.086
Gender (Male/ Female)	2.68(0.63-11.52)	0.185	1.70(0.67-4.35)	0.266
Smoke history (yes, no)	0.90(0.25-3.26)	0.872	0.923(037-2.32)	0.865
COPD	1.23(0.45-3.52)	0.689	1.24(0.59-2.62)	0.566
Pathological stage				
(I/II)/ (III/IV)	4.08(1.31-12.73)	0.015*	2.9(1.39-6.07)	0.005*
Pathological type				
AC	1	-	1	-
SCC	0.73(0.28-1.90)	0.513	0.69(0.34-1.39)	0.295
others	0.95(0.23-3.85)	0.938	0.51(0.15-1.72)	0.275
PLR	1.01(1.00-1.01)	0.103	1.06(1.01-1.11)	0.023*
NLR	1.04(0.73-1.49)	0.819	0.96(0.74-1.25)	0.761

*P < 0.05 is significant

HR: hazard rate; 95% CI: 95% confidence interval;

## DISCUSSION

In this prospective observation study, the prognostic role of preoperative PLR and NLR in NSCLC patients who underwent radical lung resection were assessed. The main finding of this study is that high preoperative PLR or NLR level indicates high risk of postoperative complications in those patients. Besides, neither PLR nor NLR correlate with worse RFS and only high preoperative PLR has a significant association with worse one-year OS.

The preoperative PLR and NLR are simply, inexpensively and widely available markers to reflect systemic inflammation status [10, 17, 18]. Several recent studies have demonstrated that preoperative inflammation state is associated with a worse postoperative outcome after surgery [19–21]. Our study showed that both preoperative PLR and NLR have a positive correlation with the PPCs particularly pneumonia and atelectasis in NSCLC patients undergoing radical surgery. As immune cells, platelets can initiate and accelerate many vascular inflammatory conditions [22, 23]. It may contribute to the ischemia-reperfusion injury induced by one lung ventilation and surgical injury. Previous study also has the similar result that preoperative thrombocytosis is associated with an increased risk of major postoperative complication [20]. Our study confirms these findings and further defines the nature of the association. We demonstrated that preoperative elevated PLR was independently associated with the development of postoperative pulmonary complication in patients with radical lung cancer surgery, but not associated with the wound healing condition.

NLR increases when neutrophil dependence inflammatory response was enhanced or lymphocyte meditated immune response was reduced, which may weaken antibacterial ability and toleration of surgery stress. Yasuhiko M. reported that NLR could independently predict the development of postoperative infectious complication and lower survival after gastrectomy[24]. Many previous researches study about prognostic value of pretreatment PLR or NLR of the NSCLC patients with chemotherapy[25, 26]. Few prospective research study about the preoperative PLR and NLR on postoperative complications of NSCLC patients. Our study reported 178 NSCLC patients with radical surgery and explore the prognostic value of both PLR and NLR. Besides, a shorter postoperative hospital stay also confirmed that NSCLC patients with low preoperative PLR or NLR had better postoperative recovery. Most patients of our study did not receive chemotherapy before surgery. Our study may provide good predictive indices for NSCLC patients without chemotherapy.

The secondary objective of our study is to evaluate the prognostic effect of PLR and NLR on OS and RFS. We observed that an increased preoperative PLR was significantly associated with a higher one-year overall survival of patients with NSCLC. The same trend was observed for NLR, although without statistical differences. The insufficiency of sample and short follow-up period may account for this result. A meta-analysis demonstrated PLR could act as a significant biomarker in the prognosis of various cancers included lung cancer [27]. Our findings are in agreement with these studies. The mechanisms behind these phenomena are not clear and it may involve many aspects. It is believed that platelets can influence multistep development of tumors [22]. A clinical research of 619 patients with epithelial ovarian cancer indicated that paraneoplastic thrombocytosis fuels tumor growth [28]. As for the relationship between high NLR and worse OS, neutrophils can favor tumor development and inhibit the activity of lymphocytes and other immune cells. Neutrophils are involved in tumor progression of non-small cell lung cancer [29]. The exact mechanisms behind the prognostic implications of an elevated NLR remain to be elucidated.

Moreover, we aim to provide a novel indicator for clinicians to efficiently estimate the optimal time course for surgical treatment of NSCLC patients. The peripheral blood count test is routinely performed in all patients. The preoperative PLR and NLR is easy to obtain and come with no extra costs for patients attempt to have a radical lung cancer surgery. It can be used for risk stratification of PPCs and postoperative long-term outcome of NSCLC patients in clinical trials and clinical practices.

Our study has several limitations. First, this study was performed in a single center, which might limit the generalizability of our observation to others. Second, the sample size of our study is small. Therefore, the risk of a type 2 error cannot be excluded. Third, our study included patients with all stage (I-IV) because of the uncertainty of the tumour stage before surgery. It increased the inhomogeneity of our study population. However, PLR and NLR were distributed balance among pathological stage. Last, the postoperative clinical outcomes were only followed up for one year. However, the trend of OS was still available.

In conclusion, preoperative PLR and NLR are independent prognostic factors of postoperative complications in NSCLC patients undergoing radical lung cancer surgery. Preoperative PLR also indicate their prognosis on cancer survival. Further investigation with larger scale is required to confirm the prognostic role of NLR and PLR in NSCLC patients undergoing radical lung cancer surgery.

## MATERIALS AND METHODS

### Participants

Between December 2014 and February 2016, patients with stage I-IV NSCLC in West China Hospital of Sichuan University who were going to receive lung cancer radical thoracotomy were enrolled. The inclusion criteria included: 1) age 30-80 years; 2) American Society of Anesthesiologists (ASA) classification ≤ III; 3) elective radical lung cancer thoracotomy. The exclusion criteria referred to: 1) infection or inflammatory conditions with clinical evidence; 2) recurrence tumor; 3) combined with other malignant tumor. The present study was approved by the Ethical Committee of West China Hospital of Sichuan University. All patients signed a written informed consent before entering the study.

### Perioperative management

To avoid the influence of surgical techniques, all surgeries were performed by one same group of surgeons. As for one lung ventilation (OLV) strategy, the tidal volume was set at 6 to 8mL/kg and positive end-expiratory pressure (PEEP) was 6 to 8 cmH_2_O under a volume-controlled mode. Recruitment maneuver composed of a continuous positive airway pressure of 30 cmH_2_O for 30 seconds was repeated every 30 minute before and after OLV to avoid atelectasis [30]. A relative strict fluid protocol was performed during the whole surgery [31]. After surgery, all patients were transferred to intension care unit (ICU) with tracheal tube and use the same analgesia pump for postoperative pain relief.

### Data collections and follow up

The basic demographic data and clinical characteristics of all patients were collected. The postoperative clinical course of each patient was carefully observed and recorded daily till discharged or 30 days after surgery. Data of blood counts within 7 days before the surgery were recorded. The blood specimen were collected through veinpuncture carried out by experienced nurses[32]. The NLR was defined as the absolute neutrophil count divided by the absolute lymphocyte count and the PLR referred to the absolute platelets count divided by the absolute lymphocyte count. Moreover, a chest computed tomography (CT) scan need to be done before discharge.

The perioperative surveillance protocol was described previously. The postoperative pulmonary complications (PPCs) were defined as the presence of pneumonia, bronchopleural fistula, respiratory failure, ARDS/ALI or massive atelectasis [33]. Cardiovascular complications included arrhythmia and cardiac failure. The definition of prolonged air-leak was postoperative chest drainage tube placed more than 5 days. Hospital stay postoperative was the period from surgery day to discharge day or death day. Hospital mortality was limited within 30 days after surgery [34].

Follow-up was down every 3-month by telephone after surgery. The contents of follow-up included tumor progression, recurrence, metastasis and survival days. Progression was defined as tumor recurrence, metastasis or death. The tumor recurrence and metastasis was assessed by clinicians.

### Statistical methods

Data collection was performed using Microsoft Office Excel and all statistical analysis was carried out with IBM SPSS Statistics for Windows, version 21.0 (IBM Corp, USA). Continuous data with a normal distribution was presented as mean ± standard deviation and median (inter-quartile range, IOR) when variables were non-normal distributions. Categorical data was described by frequency and percentage. Normality of data was tested through Kolmogorov-Smirnov one-sample test. Student's t test or one-way ANOVA (Analysis of Variance) was used for continuous variables and Fisher's exact test or χ^2^ test was used for comparison of categorical data separately.

The ROC curve analysis was carried out to assess the prognostic ability of PLR, NLR. The optimal cut-off value for NLR and PLR were also available using the ROC curve. The optimal cutoff values were identified as the values that maximize the Youden index (sensitivity + specificity − 1) [35]. Survival curves were estimated with the Kaplan-Meier method. Association between the factors and prognosis were examined with univariate and multivariate Cox regression models. In all test, a *P*-values of < 0.05 (two-tailed) was considered statistically significant.
